# The effect of an external magnetic force on cell adhesion and proliferation of magnetically labeled mesenchymal stem cells

**DOI:** 10.1186/1758-2555-2-5

**Published:** 2010-02-12

**Authors:** Toshio Nakamae, Nobuo Adachi, Takaaki Kobayashi, Yoshihiko Nagata, Tomoyuki Nakasa, Nobuhiro Tanaka, Mitsuo Ochi

**Affiliations:** 1 Department of Orthopaedic Surgery, Graduate School of Biomedical Sciences, Hiroshima University, Hiroshima, Japan

## Abstract

**Background:**

As the strategy for tissue regeneration using mesenchymal stem cells (MSCs) for transplantation, it is necessary that MSCs be accumulated and kept in the target area. To accumulate MSCs effectively, we developed a novel technique for a magnetic targeting system with magnetically labeled MSCs and an external magnetic force. In this study, we examined the effect of an external magnetic force on magnetically labeled MSCs in terms of cell adhesion and proliferation.

**Methods:**

Magnetically labeled MSCs were plated at the bottom of an insert under the influence of an external magnetic force for 1 hour. Then the inserts were turned upside down for between 1 and 24 hours, and the number of MSCs which had fallen from the membrane was counted. The gene expression of MSCs affected magnetic force was analyzed with microarray. In the control group, the same procedure was done without the external magnetic force.

**Results:**

At 1 hour after the inserts were turned upside down, the average number of fallen MSCs in the magnetic group was significantly smaller than that in the control group, indicating enhanced cell adhesion. At 24 hours, the average number of fallen MSCs in the magnetic group was also significantly smaller than that in control group. In the magnetic group, integrin alpha2, alpha6, beta3 BP, intercellular adhesion molecule-2 (ICAM-2), platelet/endothelial cell adhesion molecule-1 (PECAM-1) were upregulated. At 1, 2 and 3 weeks after incubation, there was no statistical significant difference in the numbers of MSCs in the magnetic group and control group.

**Conclusions:**

The results indicate that an external magnetic force for 1 hour enhances cell adhesion of MSCs. Moreover, there is no difference in cell proliferation after using an external magnetic force on magnetically labeled MSCs.

## Background

Human mesenchymal stem cells (MSCs) are useful for autologous cell transplantation [1]. They can differentiate into several lineages, including osteogenic, chondrogenetic, or adipogenic lineages in vitro and in vivo [2-6]. Because MSCs can be obtained from patients by minimally invasive techniques, such as bone marrow aspiration, they may provide new strategies for the regeneration of human tissues. MSCs have been examined extensively as a cell source for several tissue engineering applications [7-12].

However, many procedures are technically demanding, and require proper scaffolds or growth factors. To solve these problems, we developed a novel cell or drug delivery technique with magnetic liposome or magnetically labeled cells. Delivery of bone morphogenic protein-2 to bone [13], transforming growth factor-β1 to cartilage [14], anticancer agents to a tumor [15,16] and natural killer cells to a tumor [17] resulted in the desirable accumulation of cells or drugs to the target lesion. Ochi et al. [18] reported that the accumulation of MSC-liposome complex in the full-thickness cartilage defect was demonstrated to be higher in the external magnetic force group than in the control group. Recently, it has been demonstrated that the system using arthroscopic control and an external magnetic device could deliver magnetically labeled MSCs to an osteochondral defect and could lead to a less invasive procedure for cartilage repair [19]. In the strategies using magnetically labeled MSCs and an external magnetic force, it is necessary that MSCs adhere as well as accumulate in the target area. But the ability of an external magnetic force to promote cell adhesion has not been well studied. Also the effect of a magnetic force on cell proliferation of magnetically labeled MSCs has not been reported. The purpose of this study was to examine the effect of an external magnetic force on cell adhesion and proliferation of magnetically labeled MSCs.

## Methods

Our research methods were reviewed and approved by the ethical committee of our institution.

### Cell culture

The methods of isolation and *in vitro *expansion of bone marrow-derived MSCs are well known and have been previously described. In this study, a modification of Kotobuki's culture method was used [20]. Briefly, 5 ml of bone marrow from the tibia of adult donors (average 34 years; ranging from 24 to 46 years) was aspirated with 1 ml of heparin sodium when they underwent anterior cruciate ligament reconstruction, centrifuged for 5 minutes at 1500 rpm, and the subsequent supernatant, including heparin-sodium, was discarded. The extract was resuspended in 6 ml of culture medium, composed of Dulbecco's modified Eagle medium (Gibco BRL, Carlsbad, CA, USA) containing heat-inactivated 10% fetal bovine serum (Sigma-Aldrich Corp., St. Louis, MO, USA) and 1% antibiotics (penicillin, streptomycin, and fungizone, Bio-Whittaker, Maryland, USA). 2 ml of the suspension was seeded onto 100 mm culture dishes (Falcon, BD Bioscience), and 8 ml of culture medium was added to each dish. The dishes were incubated for three weeks under a humidified atmosphere and 5% CO_2 _at 37 degree Celsius. The medium was not changed for the first 7 days. When changing the medium, the suspended cells and the supernatant were discarded, and fresh culture medium added to the dish, where the adherent cells remained. After the first change of medium, it was subsequently changed every three days. About two weeks after seeding, the cells had proliferated and reached confluence. The cells were then harvested by treatment with 0.25% trypsin and 0.02% EDTA (2.5 g/l-Trypsin/1 mmol/l-EDTA Solution, Nacalai Tesque, inc., Kyoto, Japan). To expand the MSCs, 2-3 × 10^5 ^of the harvested cells were seeded on 100 mm culture dishes. On reaching confluence again, the cells were reseeded under the same conditions. We referred to these adherent cells as MSCs.

### Labeling of MSCs with ferumoxides

MSCs were labeled overnight with 25 μg Fe/ml ferumoxides and 375 ng/ml poly- L-lysine (PLL: Poly-L-lysine hydrobromide, MW = 388 kDa, Sigma P-1524, St Louis, MO, USA) as a transfection agent. Briefly, 2.2 μl of ferumoxide stock solution (11.2 mg Fe/ml, Tanabe Seiyaku co. Ltd., Osaka, Japan) was added per ml of culture medium, without cells, and mixed well. PLL was then added at 3.75 μl/ml from a 0.1 mg/ml stock solution. The medium was mixed, and incubated for 60 minutes at room temperature with occasional gentle mixing. Labeling was initiated by removal of the medium from the adherent MSCs, and then by adding the medium containing the ferumoxides-PLL mixture. After incubation overnight in the medium, the MSCs were collected after tripsinization, and 100% of the MSCs were labeled magnetically.

### External magnetic device

**Figure 1:**
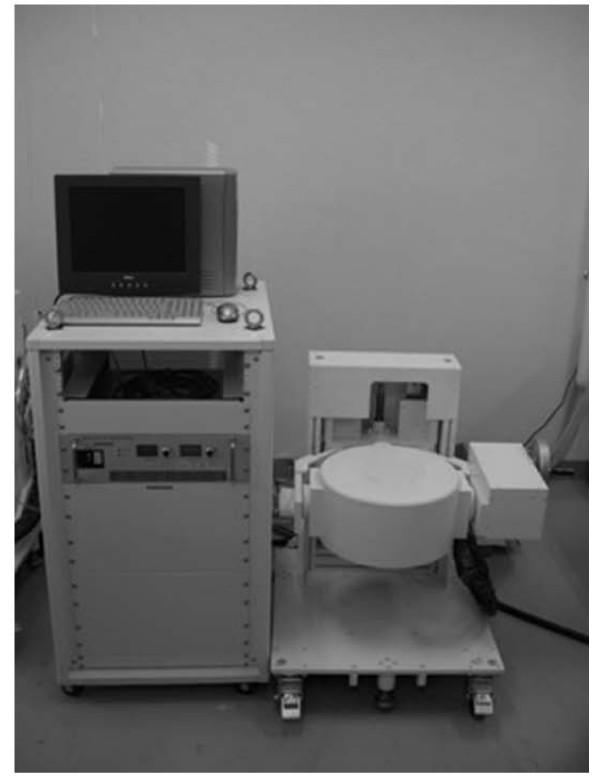
**An external magnetic device generated a magnetic force to the round wall**. The disk-shaped electromagnet consists of a solenoid iron coil and is designed to be able to generate a magnetic field efficiently. The maximum magnetic field is 0.6 Tesla.

A variable DC electromagnet (model TM-SP12010SC-014; Tamagawa Co., Miyagi, Japan) was manufactured for the purpose of generating an external magnetic force (Fig. 1). The disk-shaped electromagnet consists of a solenoid iron coil and is designed to be able to generate a magnetic field efficiently. The generating magnetic field is symmetric about the arbor of the disk. Simply, the magnetic field is directed to the center of the disk surface, and its magnitude decreases away from the surface. The magnitude of the magnetic field is increased by intensifying the electric current through the electromagnet, and is limited by the temperature of the coil. When a sample lies 8 cm from the center of the pole center, the maximum magnetic field is 0.6 Tesla, and the maximum gradient of the magnetic field's amplitude is 25 Tesla/m. The disk generating the magnet force is able to change height and direction.

### Magnetic force

**Figure 2:**
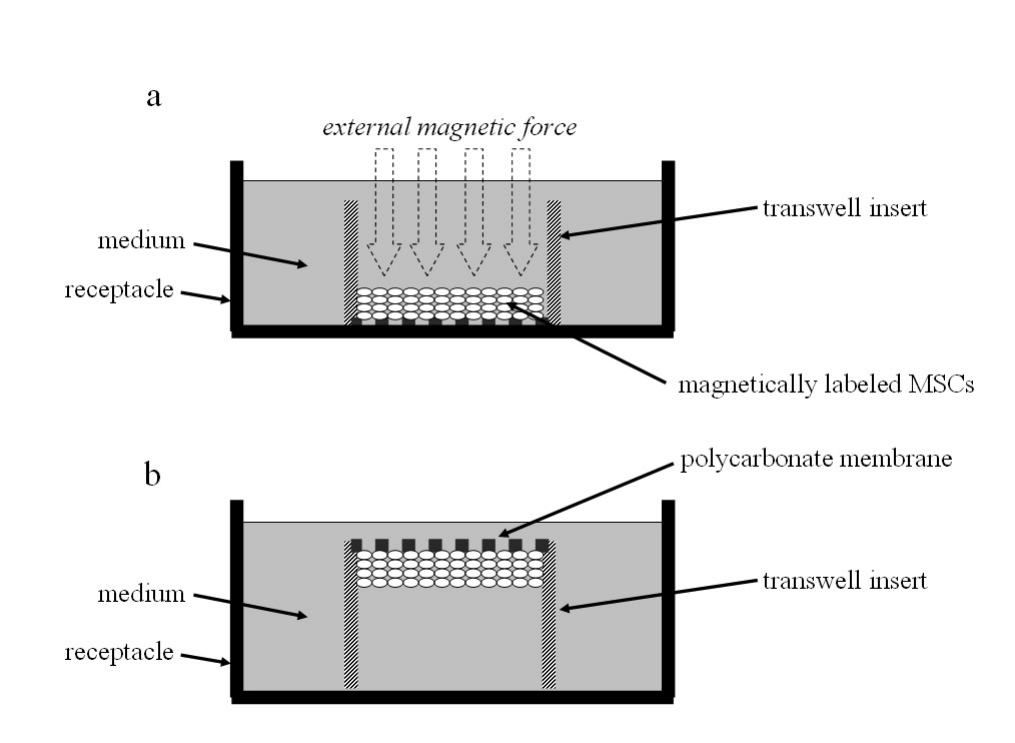
**Schematic representation of falling experiment**. (a) Magnetically labeled MSCs (1 × 10^5^) were seeded to polycarbonate membrane transwell inserts. (b) The inserts were turned upside down in the medium carefully.

Magnetically labeled MSCs (1 × 10^5^/100 μl medium) were seeded to a polycarbonate membrane transwell insert (6.5 mm membrane diameter, 0.4 μm membrane pore size, CORNING, NY, USA) (Fig. 2a). In the magnetic force group, magnetically labeled MSCs were placed under the influence of an external magnetic force (0.6 Tesla) perpendicular to the insert for 1 hour (magnetic group). In the control group, magnetically labeled MSCs were placed without the influence of a magnetic force for 1 hour (control group).

### The number of MSCs fallen from the membrane

The next step was to turn the inserts upside down in the receptacle for the medium carefully, and to place them in the incubator for between 1 and 24 hours (Fig. 2b). The number of MSCs which had fallen from the membrane was counted using a cell counter (One Cell, Hiroshima, Japan). We defined fallen cells as cells which were exist in the bottom of the receptacle for the medium after the inserts were turned upside down in the medium. The number of fallen MSCs was expressed as mean ± S.E.

### Microarray anarysis

Total RNA was extracted just after external magnetic force was affected from magnetically labeled MSCs placed under the influence of an external magnetic force using an RNeasy Plus Mini Kit (Qiagen, Hilden, Germany) according to the manufacturers' instructions. RNA was quantified by spectrometry, and the quality was confirmed by gel electrophoresis. Total RNA was converted to double stranded cDNA by the SuperScript Choice System (Invitrogen, Carlsbad, California, USA). A 15 mg aliquot of total RNA was converted into cDNA. In vitro transcription was used to produce biotin labeled cRNA from cDNA using the Ambion MEGAscript T7 kit (Ambion, Austin, Texas, USA). After fragmentation, a custom microarray was manufactured by Nimblegen (Madison, Wisconsin, USA) containing 24,000 genes using maskless array synthesis http://www.nimblegen.com. Gene chips were then scanned in a GenePix 4000B microarray scanner (Axon Instruments, Union City, California, USA). Data analysis was performed using BRB ArrayTools version 3.02 (Molecular Statistics and Bioinformatics Section, National Cancer Institute, Bethesda, Maryland, USA). Average difference values were normalized to median over the array. The data were filtered so that only those genes that were adequately measured on 75% of the arrays were included. A class comparison protocol was used to identify genes whose degree of expression differed significantly by > 1.5 fold between the two groups.

### Proliferation of magnetically labeled MSCs under the influence of a magnetic force

About 8 × 10^4 ^magnetically labeled MSCs were seeded into 60 mm tissue culture dishes and placed with or without the influence of a magnetic force for 1 hour. At various times after seeding (1, 2, and 3 weeks of culture), the number of MSCs in each dish was measured.

### Statistical analysis

The statistical significance of differences in parameters was assessed by the Mann-Whitney's U test. For all data collection, the experimenters were blinded to the group identities.

## Results

### The number of MSCs fallen from the membrane

**Figure 3:**
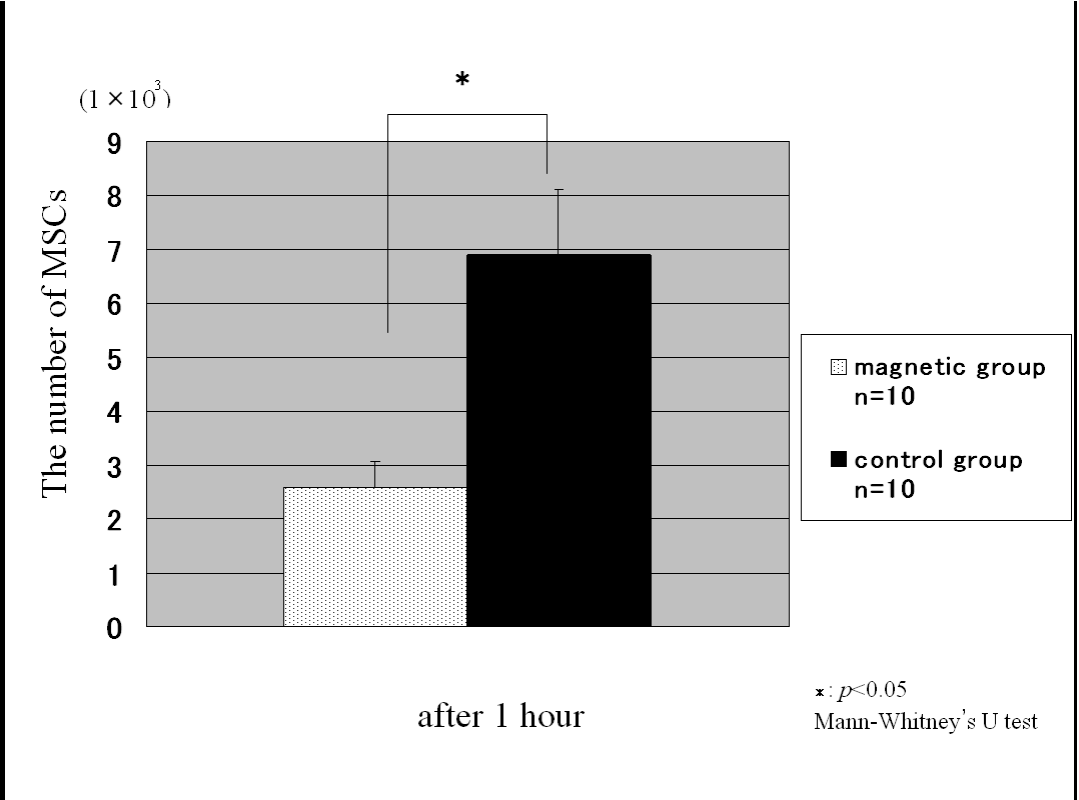
**The number of MSCs at 1 hour**. The number of MSCs which had fallen from the membrane at 1 hour after the inserts had been turned upside down. In the magnetic group, the average number of the MSCs was significantly smaller than in the control group.

**Figure 4:**
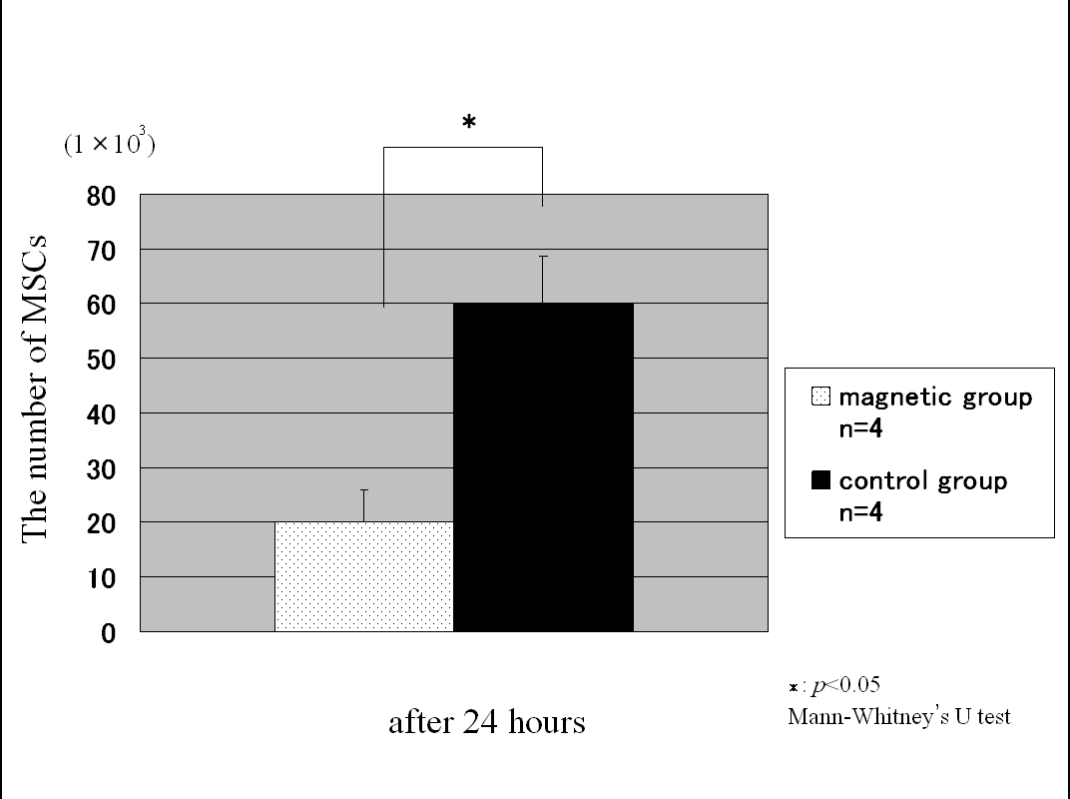
**The number of MSCs at 24 hours**. The number of MSCs which had fallen from the membrane at 24 hours after the inserts had been turned upside down. Compared with the control group, fewer MSCs were counted in the magnetic group.

At 1 hour after the inserts had been turned upside down, the average number of MSCs which had fallen from the membrane in the control group (n = 10) and in the magnetic group (n = 10) was 6.9 ± 1.1 × 10^3 ^and 2.6 ± 0.5 × 10^3^, respectively (Fig. 3). The average number of the MSCs which had fallen from the membrane in the magnetic group was significantly smaller than that in the control group. At 24 hours after the inserts had been turned upside down, the average number of MSCs which had fallen from the membrane in the control group (n = 4) and in the magnetic group (n = 4) was 6.0 ± 0.9 × 10^4 ^and 2.0 ± 0.6 × 10^4^, respectively (Fig. 4). Thus, application of an external magnetic force for 1 hour on magnetically labeled MSCs enhanced cell adhesion at both time periods.

### Microarray anarysis

The expression level and the change in expression of 24,000 individual human genes were estimated on the basis of the hybridization signal intensity of each gene-specific probe represented by the microarray system. In this study, the cell adhesion molecules were analyzed. In the magnetic group, 5 following genes were upregulated; 1.64-fold up-regulation of integrin alpha2 (ITG α2), 1.75-fold up-regulation of integrin alpha6 (ITG α6), 2.16-fold up-regulation of integrin beta3 binding protein (ITG β3 BP), 1.82-fold up-regulation of intercellular adhesion molecule-2 (ICAM-2), 2.08-fold up-regulation of platelet/endothelial cell adhesion molecule-1 (PECAM-1) (Table 1).

**Table 1:** The summary of genes upregulated in MSCs under the influence of an external magnetic force.

Gene name	Description	Fold changes
ITG A2	integrin, alpha 2 (CD49B, alpha 2 subunit of VLA-2 receptor	1.64

ITG A6	integrin, alpha 6	1.75

ITG B3BP	integrin beta 3 binding protein (beta3-endonexin)	2.19

ICAM2	Intercellular adhesion molecule 2	1.82

PECAM1	Platelet/endothelial cell adhesion molecule (CD31 antigen)	2.08

### Proliferation of magnetically labeled MSCs under influence of a magnetic force

**Figure 5:**
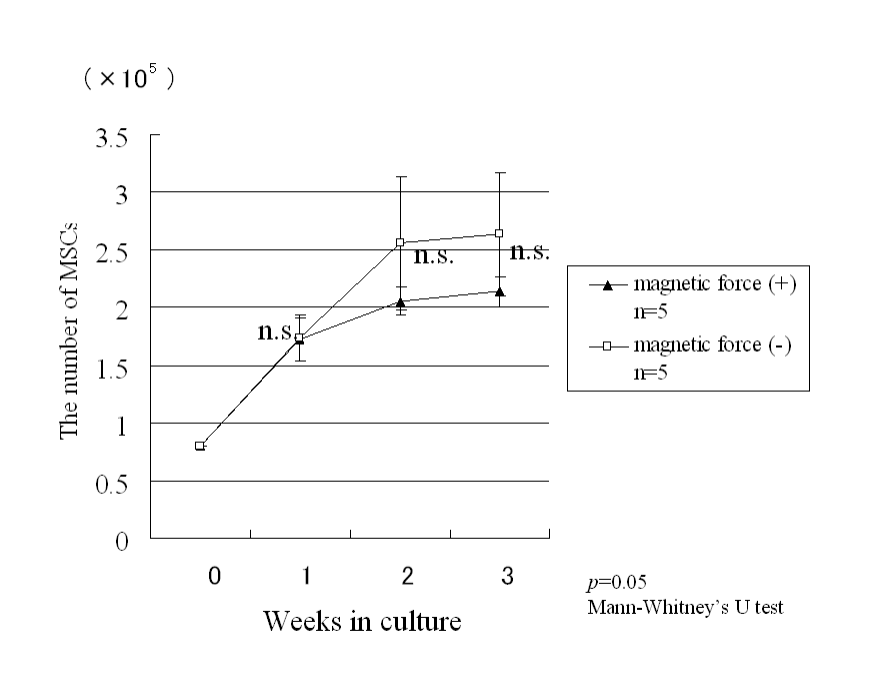
**Proliferation of magnetically labeled MSCs under the influence of a magnetic force**. After incubation for 1, 2 and 3 weeks, there was no significant difference between the number of magnetically labeled MSCs with and without the influence of a magnetic force.

After incubation for 1, 2 and 3 weeks, there was no significant difference in the number of magnetically labeled MSCs between the magnetic group and the control group (Fig. 5).

## Discussion

This study indicated that the external magnetic force increased the cell adhesion potential of magnetically labeled MSCs without affecting cell proliferation.

It is reported that MSCs are multipotent cells that can differentiate into several lineages *in vitro *and *in vivo *[2-6]. Because MSCs can be obtained easily from bone marrow, they are regarded as an important cell source that may contribute to the regeneration of human tissues. Nishimori et al. reported that intra-articular injection of MSCs along with a bone marrow-stimulation procedure repaired chronic osteochondral defects in the rat model [21]. In a clinical trial, Wakitani et al. reported that the implantation of MSCs together with high tibial osteotomy for the human osteoarthritic knee was better than only high tibial osteotomy on the arthroscopic and histologic grading scale [22]. On the other hand, Agung et al. reported that injured cartilage was repaired after intraarticular injection of MSCs in the rat model, but that an injection of too many MSCs generated free bodies of scar tissue in the joint [23]. Therefore, we conceived a new method of accumulating a relatively small number of MSCs in a desired area effectively using a magnetic force, while avoiding side effects such as the production of free bodies. Previously, we have successfully demonstrated the benefit of a cell or drug delivery system using a magnetic force. Matsuo et al. reported the efficacy of using magnetic liposomes containing human BMP and implantation of a permanent magnet at the target site of the bone or cartilage defect [13]. However, it was necessary to implant a permanent magnet into the lesion site in this procedure.

Thus, for the next step, we developed the use of an external magnetic force [16]. Ochi et al reported the efficacy of a cell and drug delivery system by a magnetic force [18]. Recently, it has been demonstrated that the system using arthroscopic control and an external magnetic device could deliver magnetically labeled MSCs to an osteochondral defect and could lead to a less invasive procedure for cartilage repair [19]. In the strategy of magnetically labeled MSCs transplantation using an external magnetic force, it is necessary that MSCs not only accumulate but also adhere to the target area. However, no reports have examined the effects on cell adhesion and cell proliferation of magnetically labeled MSCs after application of an external magnetic force.

In the present study, the average number of the MSCs which had fallen from the membrane in the magnetic group was significantly smaller than that in the control group both at 1 hour and at 24 hours after the inserts had been turned upside down. These results indicate that magnetic force increased the cell adhesion ability of magnetically labeled MSCs. There are some possibilities that magnetically labeled MSCs adhere to each other or to the membrane of the insert through cell adhesion molecules. The results of this study indicate that ITG-α2, ITG-α6, ITG-β3 BP, ICAM-2, PECAM-1 might be involved in the process of cell adhesion of magnetically labeled MSCs after using an external magnetic force. In this study, we placed magnetically labeled MSCs under the influence of an external magnetic force (0.6 Tesla) for a short time (1 hour). It is important strategy for the regeneration of human tissue to use MSCs transplantation to increase the ability of cell adhesion.

The integrin family and immunoglobulin superfamily have been known to be a members of adhesion molecules [24,25]. Integrins are the major metazoan receptors for cell adhesion to extracellular matrix proteins and, in vertebrates, also play important roles in certain cell-cell adhesions [26]. They play not only as adhesion molecule but a major role in the mediation of the cell-ECM interactions associated with structural and functional changes in surrounding tissues [27]. ITG α2 receptors bind with high affinity to collagen [26,28]. ITG α6 is a major laminin receptor [29]. ITG β3 has been implicated in a wide variety of functions, including platelet aggregation and thrombosis [30]. On the other hand, members of the immunoglobulin superfamily are membrane glycoprotein receptors containing a variable number of extracellular immunoglobulin domains.

Intercellular adhesion molecule-2 (ICAM-2) is a member of the immunoglobulin superfamily and present on leukocytes, platelets. Endothelium and platelet endothelial cellular adhesion molecule 1 (PECAM-1) is another member of the immunoglobulin superfamily expressed by leukocytes, platelets and endothelial cells. PECAM-1 molecules are particularly dense at the junctions between endothelial cells where they mainly participate to homophilic binding between adjacent cells [31,32]. In the present study microarray analysis was performed to analyze the gene expression of MSCs affected magnetic force but the immunohistochemical method or other methods were not performed to ascertain the evidence of cell adhesion. Therefore, for the limitation of the present study, it is not unclear how these adhesion molecules are involved in the process of cell adhesion of MSCs after using an external magnetic force. In the future we need to perform other studies to confirm that how these factors are involved in adhesion in the strategy using magnetically labeled MSCs transplantation and an external magnetic force.

In the present study, there are other limitations. First, it has not been confirmed whether magnetically labeled MSCs have the same differentiation capacity after the application of an external magnetic force as that without the influence of an external magnetic force. Although we have confirmed that an external magnetic force has no effect on cell proliferation, it is still necessary to research the differentiation capacity. To fully harness the potential of magnetically labeled MSCs after using an external magnetic force, further studies should be directed to ascertain their cellular and molecular characteristics. Furthermore, it has been reported by Hsieh CH et al. that 3 Tesla magnetic field for 1 hour suppressed chondrocyte growth in vitro [33]. It is also necessary to investigate whether if 0.6 Tesla external magnetic force of our study affects some characters of other cells such as chondrocyte and so on. Secondly, we used a magnetic field of 0.6 Tesla for 1 hour, but we do not know the most effective strength or duration of the magnetic field. We need to perform other studies to investigate the optimal strength or duration of the magnetic field.

Despite the fact that there were some limitations to our study, this strategy is useful and can be used clinically. Our magnetic cell targeting system is a useful tool for efficient and minimally invasive transplantation. This study may open an era of the clinical use of an external magnetic force in the future.

## Conclusions

This study indicates that an external magnetic force for 1 hour enhances cell adhesion of MSCs. Moreover, there is no difference in cell proliferation after using an external magnetic force on magnetically labeled MSCs.

## Abbreviations

MSCs: mesenchymal stem cells; PLL: poly-L-lysine; ITG-α2: integrin alpha2; ITG-α6: integrin alpha6; ITG-β3 BP: integrin beta3 binding protein; ICAM-2: intercellular adhesion molecule-2; PECAM-1: platelet/endothelial cell adhesion molecule-1.

## Competing interests

The authors declare that they have no competing interests.

## Authors' contributions

TN: participated in experiment and data collection, conducted statistical analyses, and drafted the manuscript.

NA: participated in the analysis and the interpretation of data, and participated in the revision of the manuscript.

TK: participated in the development of the study question, enrolled subjects.

YN: participated in the development of the study question, enrolled subjects.

TN: participated in the analysis and the interpretation of data.

NT: participated in the development of the study question and revision of the manuscript.

MO: conceived the main idea, participated in the design of study, and its revision and coordination.

All authors read and approved the final manuscript.
